# Capture Efficiency of Biocompatible Magnetic Nanoparticles in Arterial Flow: A Computer Simulation for Magnetic Drug Targeting

**DOI:** 10.1186/s11671-015-1127-5

**Published:** 2015-10-29

**Authors:** Thodsaphon Lunnoo, Theerapong Puangmali

**Affiliations:** Materials Science and Nanotechnology Program, Faculty of Science, Khon Kaen University, Khon Kaen, 40002 Thailand; Department of Physics, Faculty of Science, Khon Kaen University, Khon Kaen, 40002 Thailand; Nanotec-KKU, Center of Excellence on Advanced Nanomaterials for Energy Production and Storage, Khon Kaen, 40002 Thailand

**Keywords:** Magnetic drug targeting, Capture efficiency, Core/shell structure, Artery

## Abstract

The primary limitation of magnetic drug targeting (MDT) relates to the strength of an external magnetic field which decreases with increasing distance. Small nanoparticles (NPs) displaying superparamagnetic behaviour are also required in order to reduce embolization in the blood vessel. The small NPs, however, make it difficult to vector NPs and keep them in the desired location. The aims of this work were to investigate parameters influencing the capture efficiency of the drug carriers in mimicked arterial flow. In this work, we computationally modelled and evaluated capture efficiency in MDT with COMSOL Multiphysics 4.4. The studied parameters were (i) magnetic nanoparticle size, (ii) three classes of magnetic cores (Fe_3_O_4_, Fe_2_O_3_, and Fe), and (iii) the thickness of biocompatible coating materials (Au, SiO_2_, and PEG). It was found that the capture efficiency of small particles decreased with decreasing size and was less than 5 % for magnetic particles in the superparamagnetic regime. The thickness of non-magnetic coating materials did not significantly influence the capture efficiency of MDT. It was difficult to capture small drug carriers (*D*<200 nm) in the arterial flow. We suggest that the MDT with high-capture efficiency can be obtained in small vessels and low-blood velocities such as micro-capillary vessels.

## Background

In magnetic drug targeting (MDT), magnetic nanoparticles (MNPs) coated with therapeutic agents are injected into the blood vessels. Then they are vectored to specific targets by an externally applied magnetic field. This allows therapy to be concentrated in abnormal tissues while keeping therapeutic concentrations low thus reducing side effects. This can be therefore useful for treatment of cancer [1], atherosclerosis [2], arterial occlusion, stroke, and other diseases.

Magnetically responsive drug carriers can be magnetite (Fe_3_O_4_) or maghemite (Fe_2_O_3_). Their properties are related to their size. As the particle size decreases toward some critical diameter (*D*_*c*_) the formation of domain walls becomes unfavourable, and the particles are called single domain. A nanoparticle displays superparamagnetic behaviour once its size is smaller than the critical diameter [3]. That is, it exhibits net magnetization only in the presence of an external magnetic field. This allows nanoparticles (NPs) to travel freely throughout the circulatory system until they are in the presence of the magnetic field, which then acts to trap the NPs at the defined location. After removing the magnetic field, the NPs lose their previously induced magnetization thus reducing embolization in the blood vessel.

Table 1 summarizes the single-domain size of spherical particles for some common materials. They are single-domain over the size range of approximately 10–130 nm. Particles in this range are of particular interest to magnetic drug targeting. It is worth noticing that the single-domain size in Table 1 shows some large discrepancies between references [3–7], especially in magnetite. Magnetite phases may show peculiar hysteresis properties when the particle size is decreased. However, an estimate of single-domain size from hysteresis measurements [6, 7] may thus be misleading. Furthermore, critical size estimates may be more reliable if they are deduced from magnetic studies on pure magnetite samples containing a narrow range of particle sizes rather than a large size variation.

**Table 1:** Single-domain size for the MNPs below for which the material will not support a multi-domain particle

MNPs	Single domain size (nm)	Reference
Fe_3_O_4_	128	Ref. [3]
	83	Ref. [4]
	20–29	Ref. [5]
	20–30	Ref. [6, 7]
Fe_2_O_3_	166	Ref. [3]
	91	Ref. [4]
Fe	14	Ref. [3]

Fe nanoparticle is another choice for MDT [8]. It can be readily synthesized with superior magnetic properties. Nonetheless, it is not biocompatible and, typically, Fe NPs are not suitable for in vivo applications. One strategy would be to take advantage of their superior magnetic properties and enhanced hyperthermia at concentrations low enough to be nontoxic. Alternatively, a core/shell strategy to mitigate their toxicity needs to be explored. Core/shell nanomaterials are extremely important as they can have a combination of different properties and offer multifunctionality because core and shell can have different material compositions in a single particle.

**Fig. 1:**
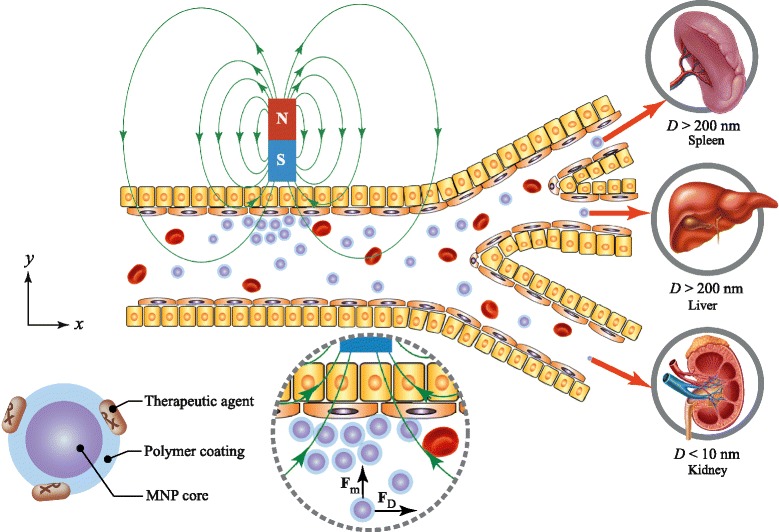
Drug-loaded carrier is typically composed of a magnetic core and a biocompatible coating material. The magnetic core was made from different materials such as Fe_3_O_4_, Fe_2_O_3_, or Fe. The coating materials are Au, PEG, or SiO_2_. Based on the biokinetics of particles, a drug carrier ranging from 10–200 nm in diameter is optimal for in vivo delivery, as the small particles (*D*<10 nm) escape by renal clearance [18] and the large ones (*D*>200 nm) are sequestered by the reticuloendothelial system of the spleen and liver [18]

The life-time of MNPs in the circulatory system is another important factor for MDT. It depends upon their size and structure. As shown in Fig. 1, the magnetic core of the drug carrier is typically coated by a biocompatible material such as gold (Au), polyethylene glycol (PEG), or silica (SiO_2_) [9, 10]. The coating layer acts to shield the magnetic particle. For example, a gold layer coated on an Fe_3_O_4_ core serves two key purposes [11]. First, it prevents oxidation of the Fe_3_O_4_ core into maghemite by forming an inert biocompatible protective coating. Second, it forms an excellent platform for conjugating drugs onto nanoparticle surfaces, since gold has a natural affinity for thiol bonds.

The primary limitation of MDT relates to the strength of the external magnetic field [12] as the magnetic gradient decreases rapidly with increasing distance to the target. A magnetic field must be applied to obtain the necessary magnetic gradient to control the residence time of NPs in the desired area. As a means to overcome limitations of using external magnetic fields, implant magnets [13] or ferromagnetic microwires [14] can be located in the vicinity of the target using minimally invasive surgery. Several studies [15, 16] have simulated the interaction between magnetic implants and magnetic NPs, enabling drug delivery. Another limitation relates to the small size of NPs, a requisite for superparamagnetism, which is needed to avoid embolization once the magnetic field is removed. Nonetheless, a small size implies a magnetic response of reduced strength. This makes it difficult to magnetically direct drug-loaded particles and keep them in the proximity of the target while withstanding the drag force of blood flow. MDT is likely to be more effective in lower blood velocities, particularly when the magnet is close to the target site. In addition, most intravenously applied NPs are recognized as “foreign” by the body system. They are immediately eliminated by macrophages of a mononuclear phagocytosis system (MPS). Generally, smaller NPs are subject to rapid elimination while larger ones show uptake by the liver, spleen, and bone marrow [17]. Based on the biokinetics of the particles, a drug-loaded carrier size range of approximately 10–200 nm in diameter is optimal for in vivo delivery, as the small particles (*D*< 10 nm) escape by renal clearance [18] and the large ones (*D*> 200 nm) are quickly eliminated by the reticuloendothelial system (RES) of the spleen and liver [18], as illustrated in Fig. 1.

The aims of this work were as follows. Firstly, the size-dependent capture efficiency of Fe_3_O_4_, Fe_2_O_3_, and Fe NPs in mimicked arterial flow was computationally studied. As the carrier size range of approximately 10–200 nm is optimal for in vivo delivery, the magnetic nanoparticle size in this range was particularly of interest. No theoretical work has so far been published on the analysis of particle size in the superparamagnetic scale as shown in Table 1. Most theoretical studies [2, 19–25] were carried out in the particle size range of 250–4 *μ*m. In fact, large particles are eliminated by the reticuloendothelial system, and they have short life-time [17] in the cardiovascular system. Secondly, the effects of three different coating layers upon the capture efficiency of MDT were investigated. The coating layers were Au, PEG, and SiO_2_. Lastly, a drug carrier structure that is suitable for MDT is introduced.

## Methods

The two-dimensional model geometrical representation of the artery with a permanent magnet is used in our model. It was assumed that the variation in transport of magnetic nanoparticles under the influence of magnetic field will be very small in the direction perpendicular to the *x*-*y* plane (see Fig. 1) because of a high aspect ratio of cross-sectional geometry that is modelled, compared with the size of a nanoparticle [26]. Moreover, a 2D model will serve as a simple, fast, and relatively accurate guideline for designing and optimizing the capture efficiency in MDT.

**Fig. 2:**
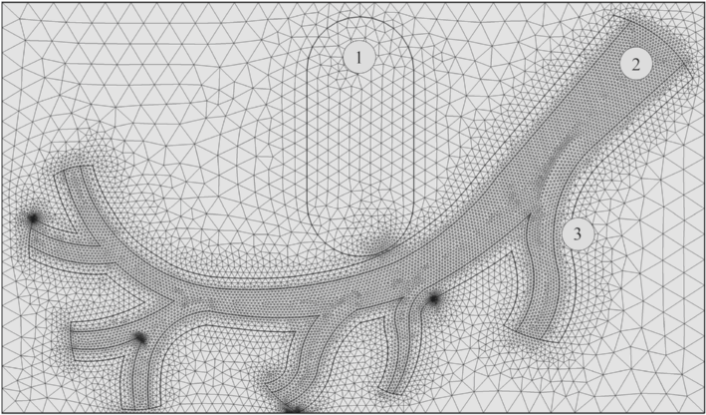
The two-dimensional model which is a representative for arterial flow. Domain (1) represents an implanted Nd-B-Fe magnet with a maximum field strength of **B** = 2 T as reported in Takeda and co-workers [44] while domain (2) and (3) are blood vessel and vessel wall of the artery. Various numerical grids in different domains are generated. The maximum element sizes for numerical mesh in domains (1), (2), and (3) were 0.083, 0.020, and 0.153 cm, respectively

The two-dimensional model for MDT is illustrated in Fig. 2. A stationary magnetic field produced by a permanent magnet implanted at a specific location is described by the following equations:

Ampère’s law

(1) $$ \nabla\times \mathbf{H}=\mathbf{J},  $$

Gauss’s law for the magnetic flux density

(2) $$ \nabla\cdot \mathbf{B}=0,  $$

and the magnetic flux density in different domains can be described by the relation between **H** and **B**

(3) $$ \mathbf{B} = \left\{ \begin{array}{ll} \mu_{0}\mu_{\text{r,mag}}\mathbf{H}+\mathbf{B}_{\text{rem}}&\text{\,\,\,magnet (domain 1)} \\[1.5ex] \mu_{0}(\mathbf{H}+\mathbf{M}_{b}(\mathbf{H})) &\text{\,\,\,blood (domain 2)}\\[1.5ex] \mu_{0}\mathbf{H} &\text{\,\,\,air and tissue (domain 3)} \end{array} \right.  $$

where **H** is the magnetic field strength (A/m), **J** is the current density (A/m^2^) and **B** is the magnetic flux density (T), *μ*_0_=4*π*×10^−7^ N/A^2^ is the magnetic permeability of air, *μ*_r,mag_ is the relative permeability of the permanent magnet, **B**_rem_ is the remanent magnetic flux density (A/m), and **M**_*b*_(H) is the magnetization vector of the blood stream (A/m), which is a function of magnetic field, **H**.

For the blood in domain 2, defining a magnetic vector potential **A** such that

(4) $$ \mathbf{B}=\nabla\times\mathbf{A} \,\,\,\,\, \text{and} \,\,\,\,\,\nabla\cdot\mathbf{A}=0,  $$

it can be shown that:

(5) $$ \mathbf{B}=\nabla\times\mathbf{A}=\mu_{0}(\mathbf{H}+\mathbf{M}_{b}),  $$

(6) $$ \frac{1}{\mu_{0}}(\nabla\times\mathbf{A})=\mathbf{H}+\mathbf{M}_{b},  $$

(7) $$ \mathbf{H}=\frac{1}{\mu_{0}}(\nabla\times\mathbf{A})-\mathbf{M}_{b}.   $$

By substituting Eq. (7) into Eq. (1), it can be found that

(8) $$ \nabla\times \left(\frac{1}{\mu}\nabla\times\mathbf{A}-\mathbf{M}_{b} \right)=\mathbf{J}.   $$

It is assumed that the magnetic vector potential has a non-zero component perpendicular only to the plane **A**_z_, which basically simplifies the 2D and it has perpendicular current equal to zero. Based on these assumption, Eq. (8) reduces to

(9) $$ \nabla\times \left(\frac{1}{\mu}\nabla\times\mathbf{A}-\mathbf{M}_{b} \right)=0.   $$

Induced magnetization of the blood, **M**_*b*_(*x*,*y*), can be explained by the arc tangent expressions [27]:

(10) $$ \mathbf{M}_{\text{bx}}=\alpha \text{tan}^{-1}\left(\frac{\beta}{\mu_{0}}\frac{\partial A_{z}}{\partial y}\right),  $$

(11) $$ \mathbf{M}_{\text{by}}=-\alpha \text{tan}^{-1}\left(\frac{\beta}{\mu_{0}}\frac{\partial A_{z}}{\partial x}\right)  $$

where *α* (A/m) and *β* (m/A) are ferrofluid magnetization-curve parameters. The material parameters *α* and *β* are obtained using M-H curve from the literature, and they are assumed to be constant for a range of magnetic nanoparticles 10–1000 nm in radius [28]. It was also assumed that the magnetic nanoparticles do not interact in the surrounding fluid. According to Taylor polynomial approximations (tan^−1^(*x*)≈*x*), it can be shown that

(12) $$ \mathbf{M}_{\text{bx}}=\frac{\chi}{\mu_{0}}\frac{\partial A_{z}}{\partial y},  $$

(13) $$ \mathbf{M}_{\text{by}}=-\frac{\chi}{\mu_{0}}\frac{\partial A_{z}}{\partial x}  $$

where *χ*=*αβ* is the magnetic susceptibility.

The flow of an incompressible non-Newtonian fluid (blood) is described by the Navier-Stokes equation. The relative importance of inertial forces compared to viscous forces is given by the dimensionless Reynolds number *Re*=*ρ*_*b*_*v*_0_*l*/*η* with *v*_0_ and *l* characteristic velocity and length scales of the blood flow under consideration. For the current study, blood density *ρ*_*b*_=1,060 kg/m^3^, the average blood velocity [23] *v*_0_≈10 cm/s, blood vessel diameter [23] *l*≈3 mm, and dynamic viscosity of blood *η*≈3.5×10^−3^ kg/(m.s), the Reynolds number *Re*≈121, which is well within the laminar flow regime. When the Reynolds number is low, the Navier-Stokes equation is

(14) $$ \rho_{b}\frac{\partial{\mathbf{u}_{b}}}{\partial t}+\rho_{b}\mathbf{u}_{b}\cdot\nabla\mathbf{u}_{b}=-\nabla p +\eta\nabla^{2}\mathbf{u}_{b}+F_{V}   $$

where **u**_*b*_ is blood velocity, *p* is the pressure, and *F*_*V*_ is the volume force (N/m^2^). The momentum transfer from MNPs to the fluid is incorporated by setting the volume force term equal to the magnetic force and the drag force. This term couples the fluid flow equation with the static magnetic field equation.

There are several models explaining the rheology of blood. The comparison of five non-Newtonian models for blood viscosity as well as a Newtonian model in the flow simulation were considered by Johnson and colleagues [29]. Given the discussion and results in Ref. [29], it was concluded that the Generalised Power Law model [30] fitted the experimental stress-strain measurements over the wide range of strain rates ($\dot {\gamma }$), $0.1<\dot {\gamma }<1000\,\,\mathrm {s}^{-1}$. The Generalised Power Law model, in addition, also encapsulates the behaviours of many of the other blood models. It is Newtonian [29] at high strain rates and behaves like the Power Law model [31] at low strain rates. Moreover, it has Casson [32] and Carreau [31] models as special cases. Therefore, the rheology of blood in our model was described using a generalized power law model [30]

(15) $$ \eta=\lambda(\dot{\gamma})|\dot{\gamma}|^{n(\dot{\gamma})-1}   $$

(16) $$ \lambda(\dot{\gamma})=\eta_{\infty}+\Delta\eta\,\text{exp}\left[ -\left(1+\frac{|\dot{\gamma}|}{a} \right)\text{exp}\left(\frac{-b}{|\dot{\gamma}|}\right) \right]   $$

(17) $$ n(\dot{\gamma})=n_{\infty}-\Delta n \,\text{exp}\left[ -\left(1+\frac{|\dot{\gamma}|}{c} \right)\text{exp}\left(\frac{-d}{|\dot{\gamma}|}\right) \right]   $$

where $|\dot {\gamma }|$ is the magnitude of the strain rate. The values used in our model were adapted from the work of Ballyk and co-workers [30]: *η*_*∞*_=0.0035 kg/(m.s), *Δη*=0.025 kg/(m.s), *a*=50 s^−1^, *b*=3 s^−1^, *n*_*∞*_=1, *Δn*=0.45, *c*=50 s^−1^, and *d*=4 s^−1^.

During the motion of MNPs in the circulatory system, there are several forces acting upon magnetic particles in viscous environments and magnetic fields. This includes magnetophoretic forces due to external magnetic fields, Stokes’s viscous drag force, Brownian force, buoyancy and gravity, inertia, and particle fluid interactions. Apart from magnetophoretic forces, Brownian and viscous drag forces, other interactions are negligible for magnetic micro- or nanoparticles. Brownian forces acting upon particle motion were considered in our model. This is due to the fact that Brownian motion and the corresponding stochastic forces significantly influence the dispersion of small particles (with *d*≤50 nm) [33, 34]. Taking these forces into account, the trajectories and velocities of the nanoparticle with mass *m*_*p*_ were calculated by integrating

(18) $$ m_{p}\frac{d^{2}\mathbf{r}}{dt^{2}}=\mathbf{F}_{M}+\mathbf{F}_{D}+\mathbf{F}_{B}.   $$

The first term on the right-hand side of Eq. (18) is the magnetophoretic force (**F**_*M*_) while the second and the third terms account for the drag force (**F**_*D*_) and Brownian force (**F**_*B*_), respectively.

Magnetophoresis is the motion of particles with respect to a surrounding medium caused by the net interaction of a magnetization with a magnetic field gradient. Magnetophoretic force **F**_*M*_ on a particle [35] is given by

(19) $$ \mathbf{F}_{M}=(\mu_{b}{m_{\text{eff}}}\cdot\nabla)\mathbf{H}  $$

where **H** is the external magnetic field, *μ*_*b*_ is the magnetic permeability of the blood, and *m*_eff_ is the magnetic dipole moment induced by the field. If we assume that particles respond linearly to the magnetic field, for spherical particles with radius *r* the magnetophoretic force [35] in a steady magnetic field is given by

(20) $$ \mathbf{F}_{M}=2\pi\mu_{b}r^{3}\mathbf{K}\nabla|\mathbf{H}|^{2}   $$

where

(21) $$ \mathbf{K}=\frac{\mu_{\mathrm{p}}-\mu_{\mathrm{b}}}{\mu_{\mathrm{p}}+2\mu_{\mathrm{b}}}.  $$

Subscripts b and p indicate blood and particle, respectively. **K** is the Clausius-Mossotti factor whose value range is −0.5≤**K**≤1.0. It provides a measure of the magnitude of the magnetophoretic force and its direction. It is called the negative magnetophoretic force if the permeability of the blood is larger than that of the particle. It is interesting to note that the magnetophoretic force is proportional to permeability of the suspension medium, the gradient of the magnetic field intensity and particle radius. Additionally, it is important to note that in our calculation it is assumed that captured nanoparticles do not influence the local magnetic field gradient.

For a particle (*Re*_*p*_≡*ρ*_*p*_|**u**_p_−**u**_*b*_|*D*/*η*≪1) having a Reynolds number so low that viscous forces dominate over inertial forces, as in generally the case for the situation studied in this work, the drag force **F**_*D*_ on spherical nanoparticles with diameter *D* is given by the Stokes drag force [36],

(22) $$ \mathbf{F}_{D}=\frac{1}{\tau_{p}}m_{p}(\mathbf{u}_{b}-\mathbf{u}_{p})  $$

where

(23) $$ \tau_{p}=\frac{\rho_{p}{D}^{2}}{18\eta}.  $$

*τ* is the particle relaxation time, **u**_*b*_ and **u**_*p*_ are blood and particle velocity, respectively.

The Brownian force was incorporated into the equation of motion. It was modelled [33, 37] as a Gaussian noise process,

(24) $$ \mathbf{F}_{B}=\zeta\sqrt{\frac{12\pi r\eta k_{B}T}{\Delta t}},  $$

where *k*_B_ is the Boltzmann constant (*k*_*B*_=1.38×10^−23^ J/K), *T* is the absolute temperature, and *Δt* is the magnitude of time step. The parameter *ζ* is a Gaussian random number with zero mean and unit variance. The random direction of the Brownian force was accounted for by evaluating both the *x* and *y* components of **F**_*B*_ at each time step using independent values of *ζ* in both dimensions.

From a magnetic drug targeting point of view, a major challenge is to create a large enough magnetic force to capture drug-loaded carrier of a reasonably small size. To optimize the capture efficiency (*ε*) of magnetic drug targeting, the class of magnetic core and coating materials must be considered. The capture efficiency for a length of artery is defined as the fraction of magnetic nanoparticles attracted by the magnetic field towards the vessel wall,

(25) $$ \varepsilon=\left(\frac{\varepsilon_{\text{in}}-\varepsilon_{\text{out}}}{\varepsilon_{\text{in}}}\right)\times 100\,\%,   $$

where *ε*_in_ and *ε*_out_ are the number of particles entering and leaving the section of artery, respectively.

The aim of our model was to mimic a more realistic situation of arterial flow, but still in a relatively simple and well-defined geometry, as illustrated in Fig. 2. Various numerical grids in different domains were generated. The maximum element size for numerical mesh in domains (1), (2), and (3) were 0.083, 0.020, and 0.153 cm, respectively. The waveform of the arterial blood flow for a reduced artery geometry was obtained by fitting a piecewise polynomial with experimental data [38]. The studied geometry included only one main inlet artery (*D*_inlet_≈4 mm) and seven outlets (*D*_outlet_≈ 0.4 – 1.5 mm). The model was characterized by two basic features occurring in blood flow during magnetic drug targeting: blood velocity profile in the artery and a non-uniform magnetic field. A schematic representation of the setup is shown in Fig. 2.

Magnetic nanoparticles were homogeneously injected at the main inlet and their trajectories were calculated by solving Eq. (18). Different sizes of nanoparticles were considered, starting from 10 nm to 2 *μ*m in radius. It is notable that the upper limit for the particle radius (*r*_max_=2 *μ*m) was determined by the characteristic size of the micro-capillary vessel. Recently, Alexiou and co-workers [13] treated squamous cell carcinoma in rabbits with ferrofluids bound to mitoxantrone (FF-MTX) that was concentrated with a magnetic field. FF-MTX was injected intraarterially (femoral artery). It was suggested by their preclinical experiment that the FF-MTX contained 6.5 mg of MTX per 10 ml. The ferrofluids consisted of iron content (*D*∼100 nm) roughly 30 mg Fe/ml (the number of particles ∼10^10^/ml). In the present model, a length of artery was studied; thus, we assumed that only 6000 magnetic nanoparticles were inserted into the arterial flow (domain 2 in Fig. 2). In order to be able to average a flow cycle, they were homogeneously distributed over the inlet at consecutive time intervals of *t*=0, 0.01, 0.02, 0.03, 0.04, and 0.05 s. The effective concentration of nanoparticles is approximately 3.15×10^−6^ mg Fe/ml.

In the human blood vessel the surface of endothelium is lined with a glycocalyx, a layer membrane bound macromolecules and adsorbed proteins. Physically, this layer is highly negatively charged [24], which interact with the moving plasma (treated as an electrolyte). Thus, the presence of the glycocalyx would possibly cause an increase in flow resistance. Pries and co-workers [39] reported that the glycocalyx layer is capable to impede plasma flow, which is probably due to high negative charge. However, Sugihara-Seki and Fu [40] suggested that the increase in flow resistance would be negligible if the glycocalyx is thin compared to the vessel diameter. Several electron microscopy studies [41] indicate the presence of the glycocalyx in the human blood vessel with a thickness of less than 100 nm, which is much thinner than the studied artery diameter (0.4 cm). The exact interaction [42] of magnetic particles with the endothelial lining is much more complicated than such a simple boundary condition does justice to. However, Haverkort and colleagues [2] showed that specific boundary conditions did not strongly influence the results. Therefore, in the present simulation, the particles were assumed to elastically collide with the arterial wall during the motion in the blood stream.

Three different magnetic cores were comparatively studied including Fe_3_O_4_, Fe_2_O_3_, and Fe. Their magnetic properties are shown in Table 2. In addition to the magnetic core, the thickness-dependent effect of three different coating layers (Au, SiO_2_, and PEG) was also carried out. The shell thickness was varied from 5–50 nm. The densities of Au, SiO_2_ and PEG were 19,320, 2648 and 1114 kg/m^3^, respectively. Once the core/shell structure was considered, the magnetophoretic force (**F**_*M*_) acting upon the particle was due exclusively to the magnetic core whereas the whole volume (core and coating layer) was taken into account for Stokes drag force (**F**_*D*_). The magnetic field originates from the implanted magnet (domain 1 in Fig. 2). The blood vessel and the vessel wall were in domains 2 and 3. The magnetic field in all domains was calculated by Eq. (1–3). The capture efficiency, defined in Eq. (25), was comparatively studied for the magnetic nanoparticles ranging from 10 nm-4 *μ*m in radius.

**Table 2:** Parameter values and properties of the materials used in this work. Note that *ρ* is density, *η* is dynamic viscosity, and *χ* is magnetic susceptibility,

Materials	Properties	Value	Unit
Blood	*ρ*_b_	1060	kg/m^3^
	*η*_b_	0.0035	kg/(m.s)
	*χ*_b_	−6.6×10^−7^	-
Fe_3_O_4_	*χ*	3.1	-
	*ρ*	5230	kg/m^3^
Fe_2_O_3_	*χ*	2.5	-
	*ρ*	4890	kg/m^3^
Fe	*χ*	3.9	-
	*ρ*	7760	kg/m^3^

All calculations in the present work were performed in COMSOL Multiphysics 4.4. For implementing the model, the following interfaces were initially required: (1) CFD module involving sophisticated blood flow models; (2) AC/DC module used for generating magnetic fields, and (3) particle tracing module which is capable of simulating particle trajectories in the blood stream. This package allowed the two-dimensional geometry of the artery and an implanted Nd-B-Fe magnet illustrated in Fig. 2 to be constructed. The governing equations used to simulate magnetic fields, non-Newtonian blood flow, and particle trajectories were Eq. (3), (14), and (18), respectively. User-defined functions were written to implement the viscosity model of Eq. (15–17) and the particle magnetophoretic force of Eq. (20). Last but not least, the capture efficiency was evaluated by Eq. (25).

## Results and discussion

### Blood flow

**Fig. 3:**
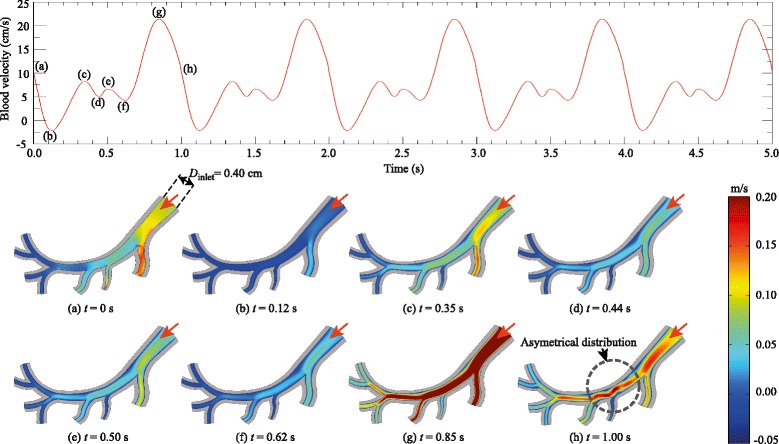
(*Top*) The physiological waveform used in this study was based on the work of Matsuo and co-workers [38], measured by means of a bidirectional Doppler flow meter catheter. (*Bottom*) The inner diameter of the inlet (*right*) is *D*_inlet_=0.40 cm. The velocity magnitude (represented by *colour bar*) in the simulated arterial flow corresponds to the velocity profile illustrated in the top figure. The *red* arrows denote the direction of flow at the inlet

After a steady-state simulation was calculated for the magnetic field in all domains, transient simulations based on the Navier-Stokes equation were performed for the arterial flow. Each cardiac cycle was from *t*=0 to *t*=1.00 s, yielding a heart rate of approximately 60 beats per minute. At the inlet, the shape of the velocity profile is a plug flow. The blood flow was observed for 5 s. Contours of the blood velocity along the artery are shown in Fig. 3. The main inlet is marked with a red arrow. The physiological waveform used in this study was based on the work of Matsuo and co-workers [38], who measured the waveform in healthy patients using a Doppler flow meter catheter. The velocity profile, obtained by digital extraction from Ref. [38] and fitting extracted data with a polynomial of degree 9, is illustrated in Fig. 3 (top). The flow pattern in the arterial flow is characterized by a small forward flow during systole (S wave) with a large forward flow during diastole (D wave). In this particular case, the peak of the S wave is just below half of the D wave. It is notable that (a) *t*=0 s is the beginning of each cardiac cycle, just prior to the deceleration of the blood flow, (b) *t*=0.12 s is the point of maximum reverse velocity, (c) *t*=0.35 s is the peak of the S wave, (d) *t*=0.50 s is halfway through the rapid acceleration phase and (e) *t*=0.85 s is the point of maximum forward velocity, the peak of the D wave. An essential aspect of the structure of the artery is its characteristic curve. For a given section of the artery structure, there occur differences, often significant, in inner diameters (left to right), and thus in flow fields. Such variations in inner diameters do not exceed the range of 2–6 mm. As shown in Fig. 3 (bottom), the inner diameter of the inlet (right) is *D*_inlet_=0.40 cm. The inner diameters of the branches (from right to left) are 0.15, 0.04, 0.11, 0.08, 0.10, 0.10, 0.10 cm, respectively. The asymmetrical distribution of the velocity profile shown in Fig. 3 (bottom) is due to the anatomical differences between the left and the right sides of the artery.

To portray complex flow patterns, the blood flow coloured by velocity magnitude in the blood vessel are shown in Fig. 3 (bottom). Side-by-side comparison of the velocity waveform and velocity contour plot at characteristic locations can be made. Figure 3 shows a representative data set of the velocity in the artery using the Generalised Power Law blood viscosity model [30]. Velocity magnitudes are shown at times, *t*= 0, 0.12, 0.35, 0.44, 0.50, 0.62, 0.85, and 1.00 s through the cardiac cycle. These values were chosen as they are representative of key points during the cardiac cycle. It can be seen from Fig. 3 (top) that the minimum and maximum values are located at *t*=0.12 s and *t*=0.85 s, in accordance with the contour plots shown in Fig. 3 (bottom).

### Particle trajectory

**Fig. 4:**
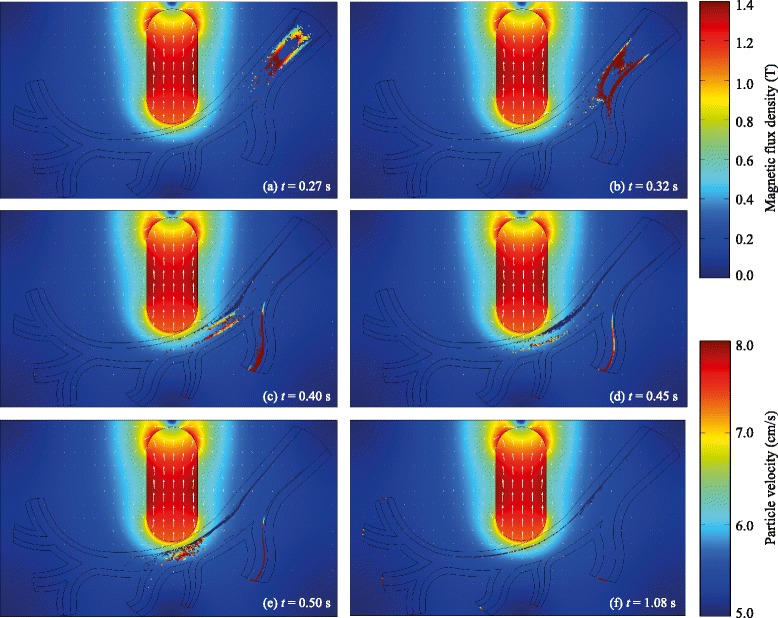
The distribution of particles (*r*_p_=2 *μ*m) after the first injection at (**a**) *t*=0.27 s, (**b**) *t*=0.32 s, (**c**) *t*=0.40 s, (**d**) *t*=0.45 s, (**e**) *t*=0.50 s, (**f**) *t*=1.08 s. The magnetization vectors are shown by the *white* arrows. The magnetic flux density and the velocity magnitude of the particles are represented by the *colour contour*. It is worth noting that the particles leaving the first branch were not included in the capture efficiency calculation

In order to mimic targeted drug delivery, an externally implanted superconductive magnet was introduced. The origin, orientation, and strength of the imposed magnetic field can be easily manipulated to cover the desired location. As can be seen from Fig. 4, the magnetic field strength rapidly decreased with increasing distance from the magnet. At the target location, the average magnetic field strength was approximately 0.5 T and the gradient of the magnetic field at the vessel centre is 1.80 T/cm. The distribution of particles along the arterial vessel is also shown in Fig. 4. The velocity magnitude of drug carrier is represented by their colours. Red represents particles moving at high velocity whereas low velocity is coded by blue. There are large holes in the particle cloud near the centre of the artery model (particularly near *t*=0.27 s and *t*=0.32 s). The large holes in the particle cloud near the centre of the artery are due to the fact that MNPs were distributed over the inlet at consecutive time intervals of *t*=0, 0.01, 0.02, 0.03, 0.04, and 0.05 s. In addition, at the inlet, the shape of the velocity profile is a plug flow. It can be clearly seen that the particles were more concentrated at the arterial wall near the externally magnetic field source while other regions remained free of the magnetic nanoparticles, confirming enhanced deposition due to imposed magnetic field.

### Particle capture

**Fig. 5:**
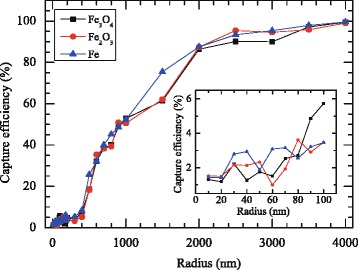
The capture efficiency (*ε*) of magnetic drug targeting in the simulated arterial flow as a function of core size. The upper limit for the particle radius was determined by characteristic size of the micro-capillary vessels (*r*_max_=2 *μ*m). The *inset* shows the efficiency of MDT in the nanoscale regime (10–100 nm)

To introduce a more objective qualification of the particle capturing, the contour of the local particle deposition for *r*_*p*_=2 *μ*m class of particles is illustrated in Fig. 4. It can be seen that for the magnetically active case, MNPs were captured at the target site. Figure 5 shows the calculation result for the capture efficiency (*ε*) of magnetic drug targeting in the simulated arterial flow as a function of core size. The size-dependent capture efficiency of Fe_3_O_4_, Fe_2_O_3_, and Fe nanoparticles (*r*_*p*_=10 –4000 nm) in mimicked arterial flow was comparatively studied. The first observation from Fig. 5 is that bigger particles have better total cumulative capturing efficiency compared to smaller particles. This is what was theoretically expected as the magnetophoretic force increases as F_M_∝*r*^3^ (as shown in Eq. (20)). For particles bigger than 2 *μ*m in radius, high capture efficiencies of 90–95 % were observed. However, the upper limit for the particle diameter is determined by the characteristic size [43] of the micro-capillary vessel (*D*_capillary_≈4 *μ*m). Therefore, we are particularly interested in particle sizes below this characteristic size.

As mentioned in the introduction, a carrier size of 10–200 nm in diameter is appropriate for in vivo delivery since larger particles (*D*>200 nm) are eliminated by the reticuloendothelial system, and they also have short life-time [17] in the cardiovascular system whereas the small particles (*D*<10 nm) escape by renal clearance [18]. Additionally, carrier sizes of 10–200 nm in diameter displays superparamagnetic behaviour thus reducing embolization when the external magnetic field source is removed. However, the capture efficiency of the drug carrier in nanoscale regime is relatively low, as shown in the inset of Fig. 5. It is clear that only a small fraction of particles is captured at the moment when the particles flow for the first time past the implanted magnet. However, the particles will flow by the magnet again, as they circulate continuously in the bloodstream. Thus, the overall capture efficiency, in the limit of many passes, could be much higher than the value shown in Fig. 5. Nonetheless, there are biological processes competing with the magnetic capture process, and this means that the overall capture efficiency will be smaller than otherwise. For particles with 50 nm in radius, 3251 nanoparticles passed through the magnetic field and only 60 particles were captured (capture efficiency=1.85 *%*) at the desired location. This small capture efficiency is due to the small magnetophoretic force. The Stokes drag force also dominates in the small particles due to high blood velocity compared to the blood velocity in smaller vessels. Apart from this, Brownian motion and the corresponding stochastic force significantly influence the dispersion of the small particles (with *d*≤50 nm) [33, 34]. The fluctuation of capture efficiency shown in the inset of Fig. 5 is due particularly to the Brownian motion of small particles.

**Fig. 6:**
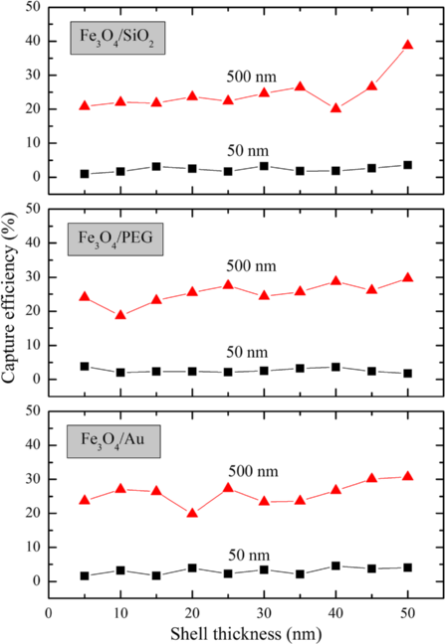
The capture efficiency (*ε*) of Fe_3_O_4_ nanoparticles coated with three different biocompatible materials including Au, PEG, and SiO_2_. The thickness of coating materials was varied from 5–50 nm. The capture efficiency of two magnetic core sizes is compared. Note that *black squares* and *red triangles* represent the core size 50 and 500 nm in radius, respectively

From a drug-targeting application point of view, we focused on a more detailed analysis of different classes of core/shell structures with different shell thicknesses. The capture efficiency of the core/shell structure is dependent upon the size of the magnetic core. Results shown in Fig. 5 can also be seen in Fig. 6. It is notable that coating Fe_3_O_4_ nanoparticles with different classes of materials (Au, SiO_2_, and PEG) did not significantly influence the capture efficiency. Once a magnetic particle is passivated by biocompatible materials (non-magnetic materials), the magnetophoretic force (**F**_*M*_) is not changed compared with the particle without passivation. In summary, the coating layer thickness did not significantly influence the capture efficiency of magnetic drug targeting as shown in Fig. 6. Similar results were found in the Fe_2_O_3_ and Fe nanoparticles.

## Conclusions

The size-dependent capture efficiency of Fe_3_O_4_, Fe_2_O_3_ and Fe NPs in mimicked arterial flow was computationally studied. Carrier sizes of 10 nm- 4 *μ*m in radius were considered. Particles larger than 2 *μ*m were efficiently captured at the desired location by the external magnetic field, and the capture efficiency was approximately 95 %. However, particle sizes in this region are not suitable for in vivo delivery as larger particles are eliminated by the reticuloendothelial system, and they also have short life-times [17] in the cardiovascular system. The suitable size is 10–200 nm in radius, but the capture efficiency of small particles decreased with decreasing size. It was found that the capture efficiency of small particles in arterial flow was less than 5 %. This is due to the reduced magnetophoretic force with decreasing size. Additionally, coating the NPs with different classes of non-magnetic materials (Au, SiO_2_, PEG) did not significantly influence the capture efficiency of MDT. In light of this, we propose that the magnetic drug targeting with high-capture efficiency will be obtained in small vessel and low blood velocity such as micro-capillary vessels.
